# Defects in Stratum Corneum Desquamation Are the Predominant Effect of Impaired ABCA12 Function in a Novel Mouse Model of Harlequin Ichthyosis

**DOI:** 10.1371/journal.pone.0161465

**Published:** 2016-08-23

**Authors:** Lei Zhang, Michael Ferreyros, Weiguo Feng, Melanie Hupe, Debra A. Crumrine, Jiang Chen, Peter M. Elias, Walter M. Holleran, Lee Niswander, Daniel Hohl, Trevor Williams, Enrique C. Torchia, Dennis R. Roop

**Affiliations:** 1 Department of Dermatology and Charles C. Gates Center for Regenerative Medicine, University of Colorado Anschutz Medical Campus, Aurora, CO, United States of America; 2 Department of Craniofacial Biology, University of Colorado Anschutz Medical Campus, Aurora, CO, United States of America; 3 Department of Dermatology, School of Medicine, University of California San Francisco, and Veterans Affairs Medical Center, San Francisco, CA, United States of America; 4 Department of Pathology and Dermatology, Stony Brook University School of Medicine, Stony Brook, NY, United States of America; 5 Department of Pediatrics, University of Colorado Anschutz Medical Campus, Aurora, CO, United States of America; 6 Service de Dermatologie et Vénéréologie, Hôpital de Beaumont, Université de Lausanne, Lausanne, Switzerland; Centre National de la Recherche Scientifique, FRANCE

## Abstract

Harlequin Ichthyosis is a severe skin disease caused by mutations in the human gene encoding ABCA12. Here, we characterize a novel mutation in intron 29 of the mouse *Abca12* gene that leads to the loss of a 5’ splice donor site and truncation of the *Abca12* RNA transcript. Homozygous mutants of this *smooth skin or smsk* allele die perinatally with shiny translucent skin, typical of animal models of Harlequin Ichthyosis. Characterization of *smsk* mutant skin showed that the delivery of glucosylceramides and CORNEODESMOSIN was defective, while ultrastructural analysis revealed abnormal lamellar bodies and the absence of lipid lamellae in *smsk* epidermis. Unexpectedly, mutant stratum corneum remained intact when subjected to harsh chemical dissociation procedures. Moreover, both KALLIKREIN 5 and -7 were drastically decreased, with retention of desmoplakin in mutant SC. In cultured wild type keratinocytes, both KALLIKREIN 5 and -7 colocalized with ceramide metabolites following calcium-induced differentiation. Reducing the intracellular levels of glucosylceramide with a glucosylceramide synthase inhibitor resulted in decreased secretion of KALLIKREIN proteases by wild type keratinocytes, but not by *smsk* mutant keratinocytes. Together, these findings suggest an essential role for ABCA12 in transferring not only lipids, which are required for the formation of multilamellar structures in the stratum corneum, but also proteolytic enzymes that are required for normal desquamation. *Smsk* mutant mice recapitulate many of the pathological features of HI and can be used to explore novel topical therapies against a potentially lethal and debilitating neonatal disease.

## Introduction

Harlequin Ichthyosis (HI, OMIM 242500) is one of the most severe recessive congenital skin diseases [1–3]. Affected infants develop large, armor-like skin plates separated by deep fissures. Although these skin plates are hard and thick, they are ineffective as a permeability barrier [4]. The hard skin plates constrict body movements, and cause the malformation of ears, eyelids and lips during development [5]. The barrier defects and the deep fissures lead to excessive water and heat loss, and render HI patients more susceptible to environmental insults [6]. Even with improvements during intensive perinatal care, many HI infants die soon after birth [7, 8].

All cases of HI caused to date have been associated with mutations in *ABCA12* (ATP-binding cassette (ABC), sub-family A, member 12), a gene that belongs to the ABC family of transporters [9–12]. The A subfamily of the ABC transporters consists of at least eleven members in humans and mice that are important for transporting various lipids in different tissues. Moreover, mutations of other A type subfamily members (e.g., ABCA1, 3, and 4) have been implicated in several human diseases with abnormal lipid metabolism [13–15]. The known roles of ABCA proteins in lipid transport have led to a focus on abnormal lipid metabolism in the epidermis as the primary cause of the HI pathology [16, 17].

Studies of human HI patients have revealed approximately 50 independent *ABCA12* mutations that can lead to HI. These occur throughout the coding region and often result in protein truncation due to nonsense mutations, although there are also a number of splice site mutations that affect exon usage, as well as missense mutations. Importantly, mutations in *ABCA12* have been associated with Type II Lamellar Ichthyosis (LI 2, OMIM 601277) and Non-Bullous Congenital Ichthyosiform Erythroderma (NBCIE, OMIM 242100) [18–20], both less severe forms of the disease.

Several lines of evidence have led to the hypothesis that ABCA12 plays a critical role in packaging glucosylceramides (GlcCer) into intracellular lamellar bodies (LBs) that are then delivered to the interstices of the stratum corneum (SC), allowing for “cargo” lipids to be processed and incorporated into the intercellular lipid barrier. First, ABCA12 colocalizes with LBs [21]. Second, LBs appear abnormal and there is a the lack of extra-cellular lipid lamellae in HI skin [16, 22]. Third, GlcCer is mislocalized in HI keratinocytes in vitro, which can be corrected by *ABCA12* gene transfer [10]. This hypothesis has gained further support from several *Abca12* mutant mouse models that recapitulate many of the pathological features of HI, including strikingly thickened SC, skin fissures, trans-epidermal water loss, limb contractures, and facial malformation [23–25]. Lipid analysis of the skin from these mice suggests that ABCA12 transporter is critical for GlcCer trafficking during epidermal barrier formation [23, 25].

Here we describe a new recessive mouse model, “*smooth skin*” or “*smsk*”, derived from an N-ethyl-N-nitrosourea (ENU) mutagenesis screen that displays many of the hallmarks of HI. Analysis of this *Abca12*^*smsk/smsk*^ strain further reveals that the ABCA12 transporter is involved in two critical processes essential for normal desquamation: the transfer of lipids and the transport of proteolytic enzymes to the SC. Lastly, we show that topical delivery of exogenous KALLIKREIN (KLK) proteases to the *smsk* mouse model can increase desquamation and ameliorate the HI phenotype. This suggests a potential new treatment for human HI patients.

## Results

### Identification of a recessive mutant mouse line with skin defects

**Fig 1:**
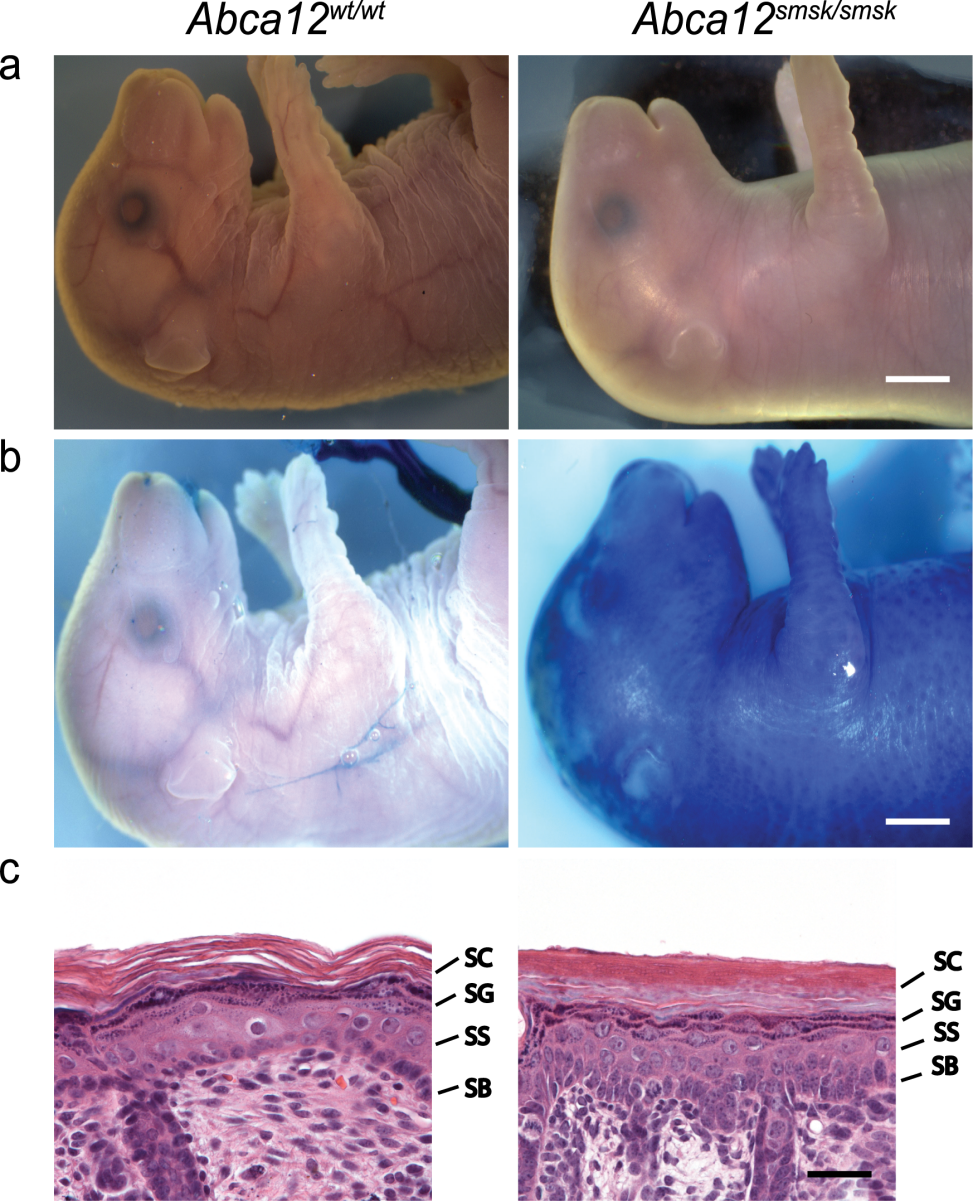
Skin abnormalities in *smsk* mice. (a) E18.5 *smsk* embryos display a shiny, tight and thickened skin phenotype with limb contractures. (b) *Smsk* E18.5 embryos have epidermal barrier defects as demonstrated by a toluidine dye penetration assay. (c) Histology of mutant E18.5 embryonic skin demonstrates severe hyperkeratosis with more than 16 layers of corneocytes in the SC and disorganized architecture in other epidermal layers. Bar for a and b = 2.5 mm. Bar for c = 25 μm. Epidermal layers: SB = stratum basale; SS = stratum spinosum; SG = stratum granulosum; SC = stratum corneum.

Following a recessive screen based on ENU-treated founder C57BL/6J male mice and a three-generation cross we identified a mutant line we named s*mooth skin (smsk)*, in which 25% of the offspring (120 of 498) presented with a pronounced perinatal lethal skin phenotype. Development of the defective skin condition was studied by examining *smsk* homozygous mutants at different embryonic stages. Mutant embryos showed a normal appearance at E14.5, but at E16.5 mutants developed a partial absence of normal skin folds around the trunk and limbs, and by E18.5 the mutant embryos developed a taut, thick skin and limb contractures (Fig 1A). Newborn mutant pups died within a few hours after birth, and appeared severely dehydrated with dry cracking skin (data not shown).

We performed toluidine dye penetration assays on embryos at E18.5 when the skin barrier normally becomes fully functional, just prior to birth, to test whether the permeability of *smsk* skin was altered. The mutant skins stained entirely blue, whereas skins of wild type (WT) littermates were impenetrable to the dye (Fig 1B), demonstrating a defective skin barrier underlying the *smsk* phenotype. Histologically, skin sections from *smsk* embryos at E18.5 lacked the loose pattern of the normal SC, revealing instead a hyperkeratotic epidermis. Transmission electron microscopy (TEM) showed that mutant SC contained twice as many layers as WT skin (16 ± 1.8 vs. 8 ± 2.6 (Mutant vs WT, Avg ± SD), p<0.0003). Although all epidermal cell layers were present in mutant epidermis, there was an expansion of the granular layer, a reduction in the size of the spinous layer, and a disorganized basal layer (Fig 1C). Despite the gross morphological skin defects, the hair follicles appeared to develop and differentiate normally.

### Genetic Mapping Indicates that *smsk* is caused by a splice site mutation in *Abca12*

**Fig 2:**
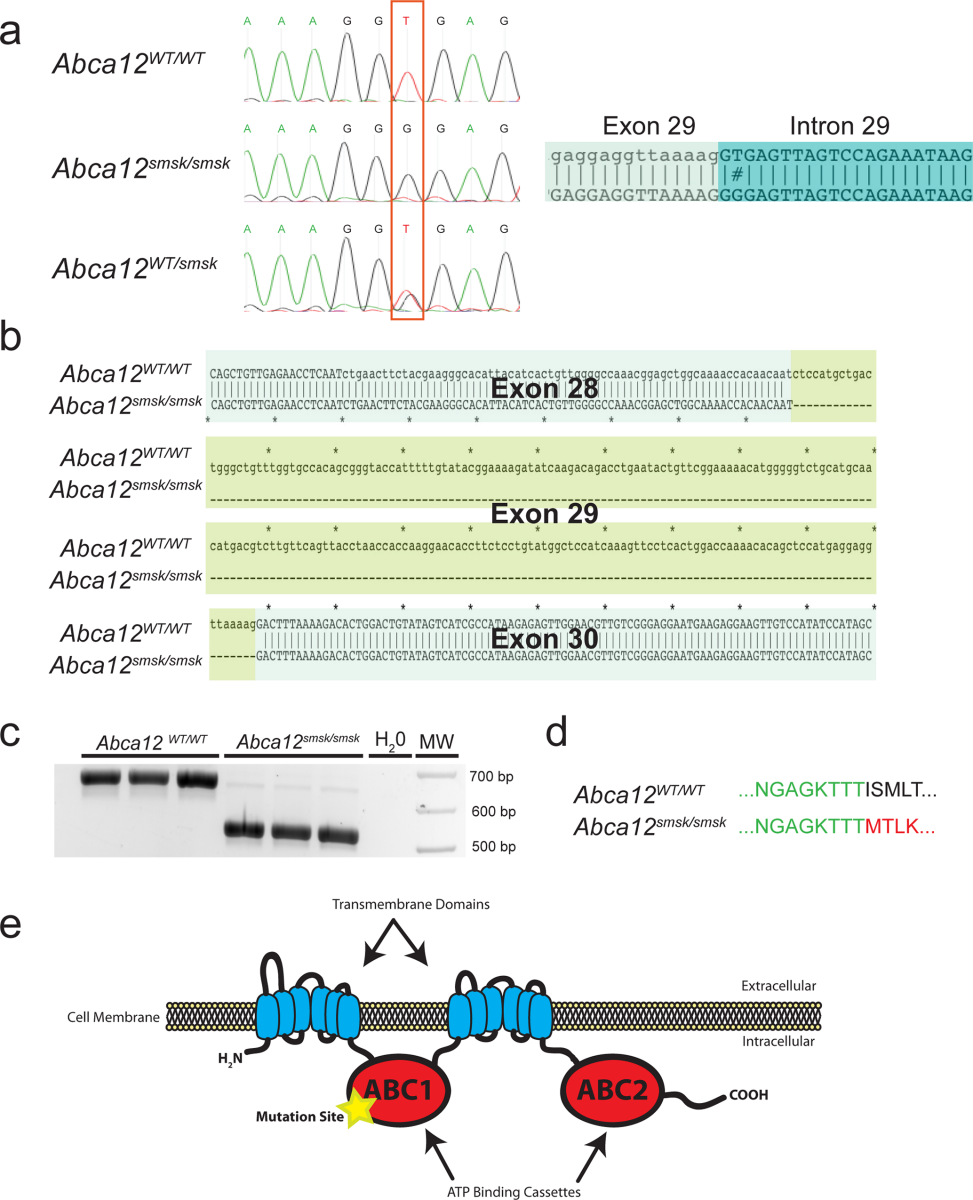
A mutation in *Abca12* is associated with the *smsk* phenotype. (a). The recessive mutation responsible for the skin phenotype of *smsk* was mapped by linkage analysis to a region containing the *Abca12* genomic locus. Genomic DNA sequencing showed that the wild type (WT) 5’ splice donor site “GT” in the 29th intron of *Abca12* on the C57/B6 background was mutated to “GG” in the phenotypic mice. Non-phenotypic carrier mice contained both “GT” and “GG” alleles. (b) Alignment of WT and mutant *Abca12* cDNA sequences showed that the splice site mutation resulted in a skipping of exon 29 in the mutant *Abca12* mRNA and would produce an in-frame deletion of amino acids encoded by exon 29 in the mutant protein. (c) The cDNA fragments of *Abca12* encompassing exon 28 to 31 were amplified by RT-PCR from skin RNA of WT (764bp) and *smsk* mutant (HI, 545bp) mice. The bands were resolved by agarose gel electrophoresis. (d) In silico translation analysis predicted that the *smsk* mutation results in a truncated protein with an in-frame fusion between amino acid sequences encoded by exons 28 (green) and 30 (red). The normal sequence encoded by the splicing of exon 28 to exon 29 is shown in black. (e) Alternative splicing skips exon 29, leading to an in-frame deletion of 73 amino acids from the first ATP-binding domain of ABCA12.

Genetic mapping of the *smsk* mutation was initially accomplished by outcrossing the ENU-mutagenized C57BL/6J founder male onto a WT 129S1/SvImJ background. Analysis of MIT microsatellite markers followed by deep sequencing revealed that the mutation responsible for the skin phenotype was in a 5’ splice donor site in intron 29 of *Abca12* (Fig 2A). The mutation causes a thymine to guanine transversion at the second nucleotide of the downstream intron and converts the consensus donor sequence ‘AGGT’ to ‘AGGG’ (Fig 2A). Analysis and sequencing of the *smsk* transcript between the region corresponding to exons 28 and 30 in the WT controls confirmed the deletion of exon 29 (Fig 2B and 2C). The skipping of exon 29 in the *smsk* allele would predict an in-frame deletion of 73 amino acids resulting in a truncated protein lacking a critical part of the first ATP binding cassette beginning at amino acid 1388 (Fig 2D and 2E). Hereafter, the *smsk* mutant line is referred to as *Abca12*^*smsk/smsk*^.

### Defective glucosylceramide trafficking, abnormal lamellar body and intercellular lipid lamellar structure in *Abca12*^*smsk/smsk*^ epidermis

**Fig 3:**
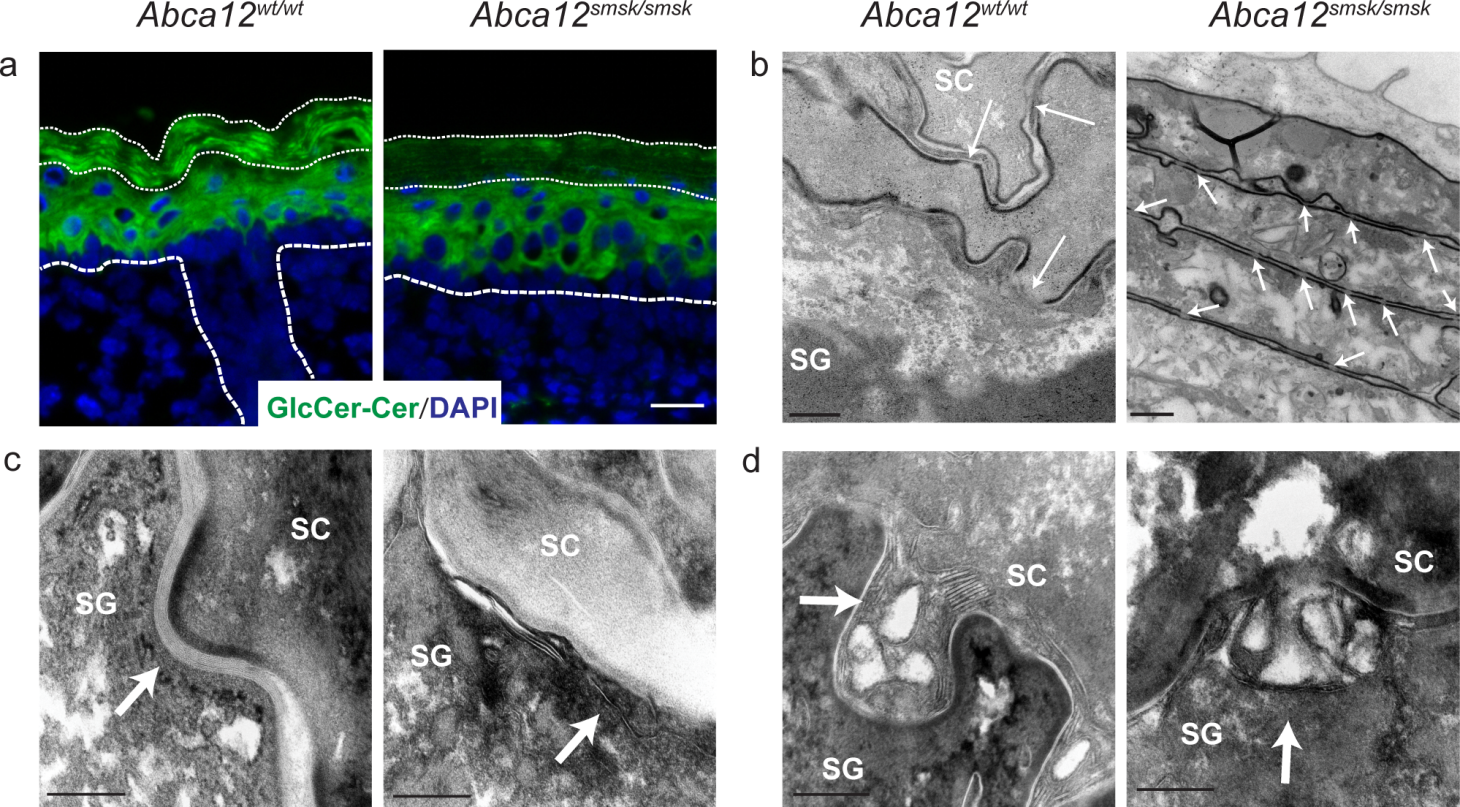
Defects in lipid accumulation in the stratum corneum of *Abca12*^smsk/smsk^ mutant mice. (a) Immunofluorescence staining for glucosylceramide/ceramide (GlcCer/Cer) showed localization throughout the suprabasal layers of WT epidermis, whereas reduced levels were detected in the SC (thin dashed lines) of E18.5 *Abca12*^*smsk/smsk*^ epidermis. Bar = 25 μm. (b) Transmission electron microscopy (TEM) shows the disappearance of corneodesmosomes (CDs) (white arrows) above the SG-SC interface in WT mice, whereas CDs are retained in *smsk* SC. Bars = 200 nm and 500 nm respectively. (c) TEM pictures show the presence of normal intercellular lipid lamellae (arrow) at the junctions between SG and SC layers in the WT epidermis but not in the mutant epidermis. Bars = 200 nm. (d) Ultrastructural analysis shows that lamellar bodies (LBs) in WT epidermis were loaded with lipid lamellae and fused with the surface of granular cells (arrow). LBs in mutant epidermis had no lamellar cargo, but fusion with the granular cell membrane appeared normal. Bars = 200 nm.

The connection between the *smsk* mutation and the lipid trafficking defects typifying HI were further explored using histological and ultrastructural analyses. Ceramides (Cer) in the SC are mainly derived from GlcCer and are the major components of the lamellar layers between the corneocytes. GlcCer molecules are synthesized by differentiating keratinocytes, packaged into LBs, and secreted into the intercellular space between the granular layer and cornified layer, where they are enzymatically processed into Cer and included in the lipid lamellae [26–29]. Immunofluorescence was employed to co-examine the distribution of GlcCer and Cer (GlcCer/Cer) in mutant skin. In WT epidermis, GlcCer/Cer staining was continuous in all epidermal cell layers, with strongest staining observed in the SC. However, mutant epidermis showed weaker GlcCer/Cer staining in both the basal and suprabasal layers, and was strikingly reduced in the SC (Fig 3A), consistent with defective GlcCer trafficking in *Abca12*^*smsk/smsk*^ epidermis.

TEM was performed to examine the gross appearance of the SC and lipid trafficking defect at an ultrastructural level. As shown in Fig 3B, WT skin showed normal lipid secretion and disappearance of corneodesmosomes (CDs) above the stratum granulosum (SG) and first two layers of SC, whereas *smsk* skin showed the collapse of adjacent cells and persistence of CDs. Enumeration of CDs revealed significantly higher density (number of CDs/length of corneocyte membrane) in both the lower and upper layers of the *smsk* SC (S1 Fig). Moreover, incomplete cornification was evident in mutant SC with cells appearing transitional. Multi-lamellar structures were apparent in the spaces between the corneocytes and cornified and granular layers of WT skin, but these structures were not observed in *Abca12*^*smsk/smsk*^ epidermis (Fig 3C). The granular cells from mutants were superficially similar to controls, with numerous structures resembling LBs observed in the cytoplasm or fused to the cell membrane (Fig 3D). However, no multilayered cargo was observed in the vesicles of *Abca12*^*smsk/smsk*^ granular cells. The corneocyte lipid envelope appeared normal in *Abca12*^*smsk/smsk*^ epidermis, a finding in common with HI patient pathology [28–30]. Overall, our data indicate that defects in lipid trafficking in the SG-SC layers are associated with the loss of exon 29 from the *smsk* transcript.

### Terminal differentiation is deregulated in *Abca12*^*smsk/smsk*^ epidermis

**Fig 4:**
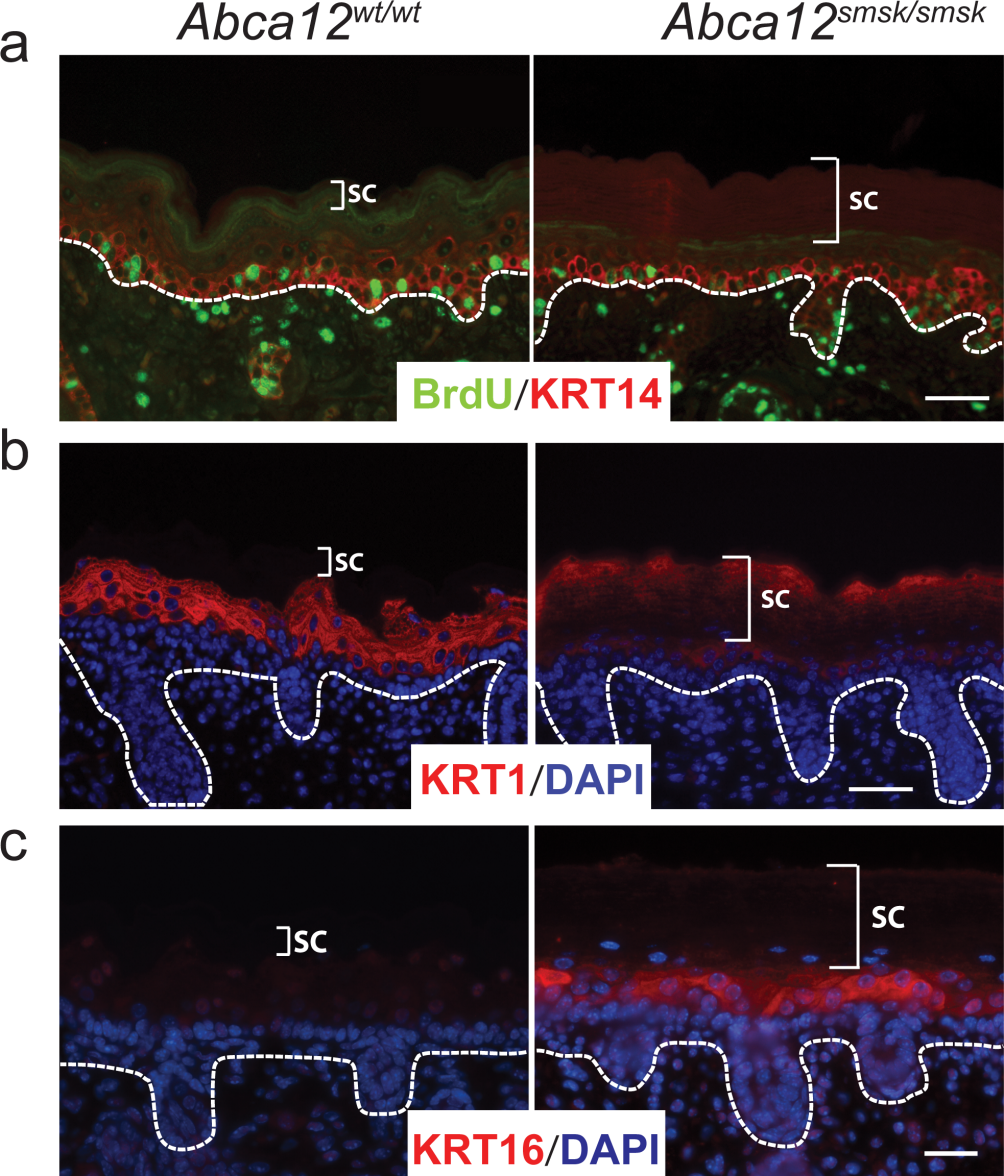
*Abca12*^*smsk/smsk*^ displays defects in terminal differentiation. (a). Cell proliferation rates were similar in both WT and *smsk* embryonic epidermis as determined by *in utero* incorporation of 5-bromo-2'-deoxyuridine (BrdU). Sections were stained with an anti-BrdU antibody (*green*) and counterstained with KERATIN 14 (KRT14) (red). Note that no difference in the expression pattern of KRT14 was observed in either WT or mutant epidermis. Bars = 25 μm. (b) Keratinocytes in *Abca12*^*smsk/smsk*^ mutant skin undergo abnormal terminal differentiation as demonstrated by scattered pattern of KRT1 (red) detection in the SC. Bars = 50 μm. (c) KRT16 (red) was ectopically expressed in the interfollicular suprabasal keratinocytes of *Abca12*^*smsk/smsk*^ epidermis, whereas it was undetecteable in the WT epidermis. Furthermore, the retention of nuclei (parakeratosis) in the SC is observable in *smsk* mutant skin. Bar = 25 μm.

To study overall development of the skin, the expression of representative differentiation markers and cell proliferation were examined in the epidermis of E18.5 embryos. Staining for the basal layer marker KERATIN 14 (KRT14) showed a comparable distribution pattern in both WT and mutant epidermis (Fig 4A). Staining for LORICRIN and REPETIN also displayed similar distribution patterns in both WT and mutant epidermis (data not shown). We also determined that epidermal proliferation, measured using an *in utero* 5-bromo-2'-deoxyuridine (BrdU) incorporation assay, was similar in the basal layer of WT and mutant fetal epidermis (Fig 4A). In contrast, a clear difference between WT and mutant skin was apparent when using an antibody that recognizes a C-terminal epitope of KRT1 that is cleaved during maturation [31]. In WT skin, immunostaining was only observed in the suprabasal layers and was not seen in the SC as the epitope is cleaved off during terminal maturation. In contrast, immunoreactivity in mutant skin extended from the spinous layers to the SC (Fig 4B). Furthermore, we observed a marked induction of the “stress” keratin, KRT16, in *smsk* epidermis, whereas KRT16 was undetectable in WT skin (Fig 4C). Furthermore, staining of KRT6, which is normally only expressed in the developing hair follicles at this age [32], was increased in the suprabasal layers of the *Abca12*^*Smsk/Smsk*^ epidermis (not shown). Induction of both KRT6 and -16 indicate that *smsk* skin was under “stress” conditions. Parakeratosis was also evident in the SC of *Abca12*^*smsk/smsk*^ mice (Fig 4C). Taken together our results are indicative of aberrant differentiation caused by the *smsk* mutation, a similar pathology to that observed in HI patients.

### Retention of desmosomal/corneodesmosomal components in *Abca12*^*smsk/smsk*^ epidermis

**Fig 5:**
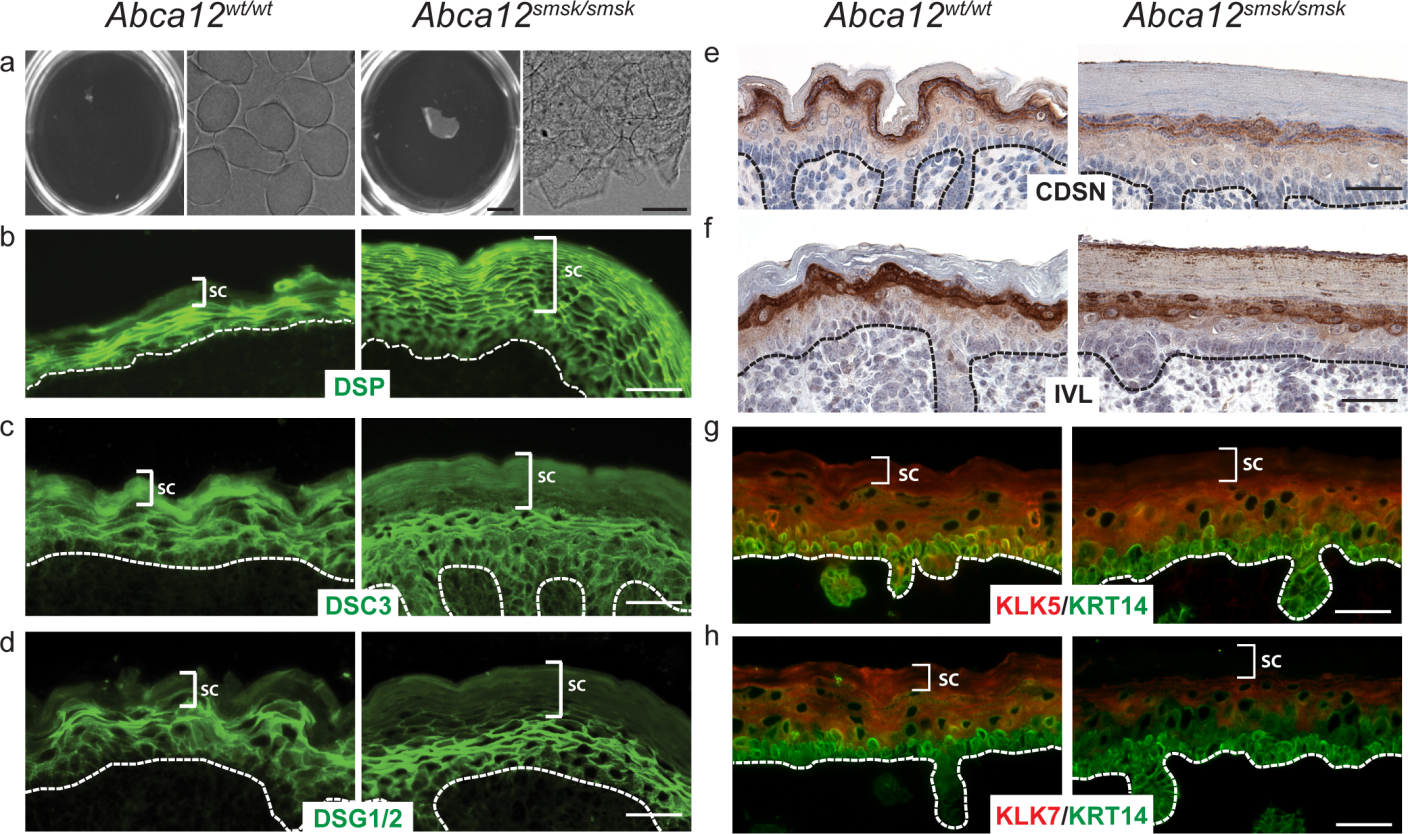
Enhanced adhesion of cornified envelopes in the *Abca12*^*smsk/smsk*^ epidermis. E18.5 skin pieces from both WT and *Abca12*^*smsk/smsk*^ embryos were boiled in cornified envelope (CE) extraction buffer. (a) WT skin pieces dissociated after boiling for 10 minutes, resulting in the dissolution of skin into individual CEs (Left panels). The mutant skins were never completely dissolved, even after extended hours of boiling. No individual CEs from the mutant skin were evident (Right panels). Bars = 5 mm and 20 μm, respectively. (b-f) Skin sections of both WT and *Abca12*^*smsk/smsk*^ embryos showed (b) expression of DESMOPLAKIN (DSP), a component of both desmosomes and CDs, persisting throughout the thickened SC of the *smsk* epidermis; (c) a minor decrease in DESMOCOLLIN (DSC3) staining in the *smsk* SC; (d) no change in DESMOGLEIN (DSC) 1&2 staining in mutant skin versus WT; (e) reduced SC localization of CORNEODESMOSIN (CDSN) in mutant skin; (f) and INVOLUCRIN (IVL) staining throughout the SC of mutant mice but reduced levels in lower layers compared to WT skin. (g-h) Immunofluorescence detection of KLK5 and -7 revealed their presence in all layers of the WT epidermis with prominent expression observed at the junction between granular and cornified layers. In contrast, KLK staining was reduced in the *smsk* SC. Bars = 25 μm.

To define the functional consequences of the *smsk* mutation, we performed a boiling dissociation assay of the SC. WT SC typically dissociates into individual cornified envelopes (CEs) upon boiling in extraction buffer (Fig 5A). In stark contrast, the SC of ABCA12 mutant skin never disassociated and instead floated as a homogenous sheet (Fig 5A). Under microscopic examination, corneocytes in mutant epidermis were still tightly attached to each other even after prolonged attempts to separate them (Fig 5A). As indicated above, the reduction of lipid secretion from SG keratinocytes in *smsk* skin likely contributed to the tight packing of mutant corneocytes. Another contribution to the observed corneocyte adherence in *smsk* SC could be biochemical changes in the desmosomal components of the *Abca12*^*smsk/smsk*^ epidermis. As desmosomes mature into CDs, CORNEODESMOSIN (CDSN) further strengthens the adhesion of adjacent corneocytes. Subsequently, these desmosomal components are gradually degraded by desquamatory proteases, such as KLK5, and -7 [33, 34]. In WT SC, the detection of the ‘fishnet’ pattern for DESMOPLAKIN, a desmosomal protein that connects desmocadherins to intracellular keratin fibrils [35, 36], was restricted to the granular layers. In contrast, in *Abca12*^*smsk/smsk*^ epidermis the DESMOPLAKIN staining extended to all layers of the thickened SC (Fig 5B). The desmosomal and corneodesmosomal cadherin DESMOCOLLIN3 (DSC3) was less apparent in *smsk* SC, whereas DESMOGLEIN 1/2 (DSG) levels and distribution remained similar to WT epidermis (Fig 5C and 5D). Staining for CDSN, which is delivered to the SC interstices by LBs [37], was markedly decreased in the SG of the mutant skin compared to WT littermates (Fig 5E). This mirrored the reduction observed in GlcCer/Cer levels (Fig 3A). The reduction of CDSN staining by immunohistochemistry was not due to reduced transcription as its mRNA abundance was elevated in *smsk* mutant skin (S2 Fig). Interestingly, *Abca12* mRNA levels were also upregulated in *smsk* skin, which may indicate feedback regulation of its expression in response to deficiency of its cellular function (S2 Fig). We also stained for INVOLUCRIN (IVL), a component of the cornified lipid envelope. IVL staining was similar in the SG of WT and mutant, but it was also detected throughout the SC layers of mutant skin and appeared membrane associated, suggestive of altered processing (Fig 5F).

### KALLIKREIN proteases are not delivered to the stratum corneum in *Abca12*^*smsk/smsk*^ epidermis

The severe hyperkeratosis, retention of corneocytes, and reduced detection of CDSN suggested that there could be a desquamatory defect in mutant epidermis. KLK5, -7 and -14 are major serine proteases that mediate desquamation and are secreted into the extracellular spaces at the junction between the granular and cornified layers, where their enzymatic activities are initiated by self-cleavage of pro-KLK5. The activated KLKs then digest CD components that bind the corneocytes together, thus allowing these cellular structures to be shed [33, 36, 38]. We examined the distribution of both KLK5 and -7 in the epidermis using immunofluorescence staining. Compared to WT in which staining could be observed in all epidermal layers, the distribution of both KLK5 and -7 decreased significantly from the upper granular layers through all levels of the SC in *Abca12*^*smsk/smsk*^ mutants (Fig 5G and 5H). Similar to *Abca12* and *Cdsn*, *Klk7* mRNA abundance was increased in mutant skin (S2 Fig), indicating that decreased immunodetection of KLK7 was not due to reduced transcription.

### KALLIKREIN proteins and labeled ceramide metabolites colocalize in differentiating keratinocytes

**Fig 6:**
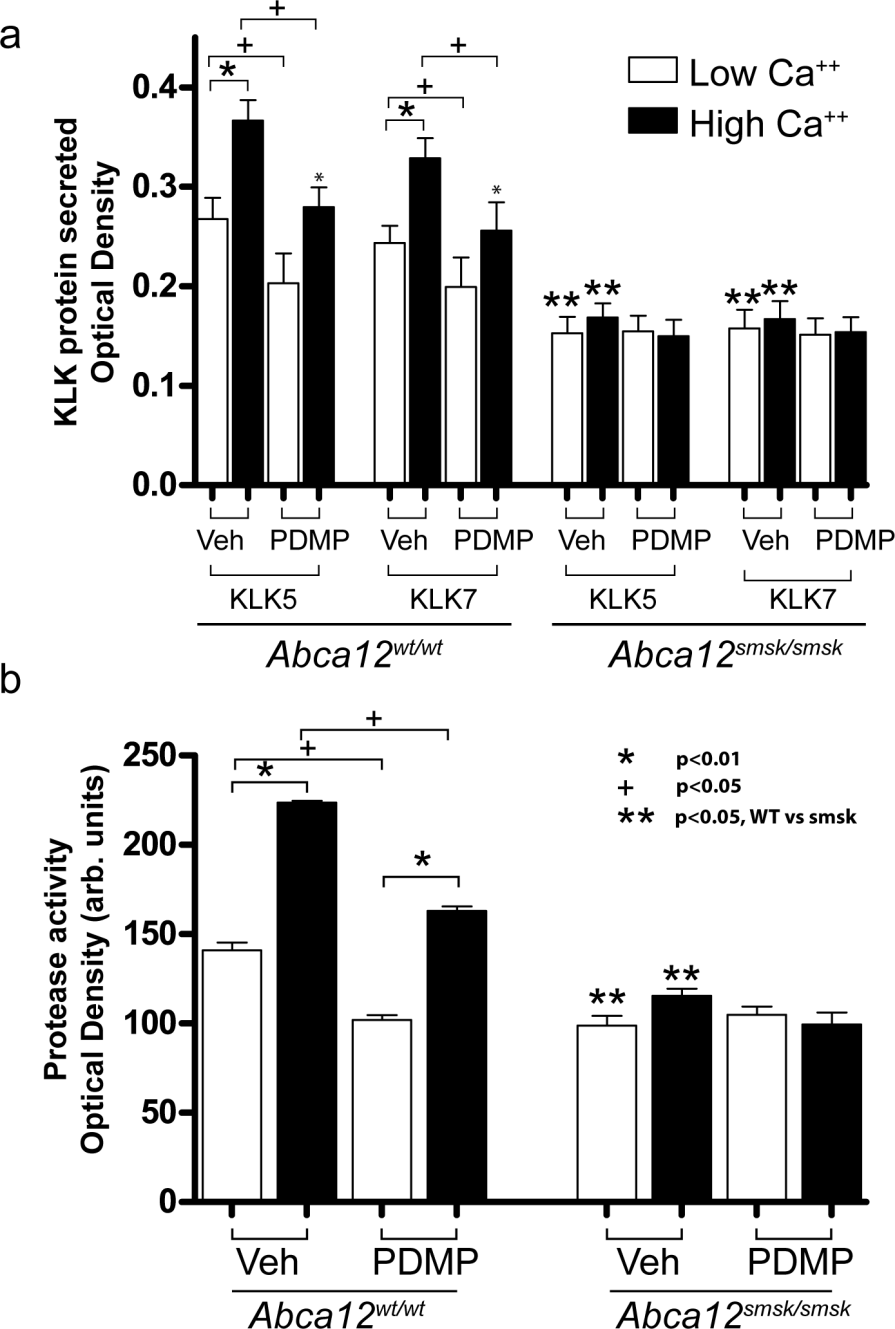
Inhibition of glucosylceramide production mimics defects in KALLIKREIN secretion from cultured *Abca12*^*smsk/smsk*^ keratinocytes. (a) Secretion of KLKs into the culture media from cultured keratinocytes were measured via ELISA. Basal levels of KLK5 and -7 were readily detected in the culture media of keratinocytes grown under low Ca^++^ conditions, and this increased after differentiation induced by elevated Ca^++^ levels (p<0.01 for both KLK5 and -7). Pre-treating WT keratinocytes with d,l-threo-1-phenyl-2-decanoylamino-3-morpholino-1-propanolhydro-chloride (PDMP) significantly reduced secreted KLK5, -7 levels (p<0.05). Note that levels of KLKs in the media of *Abca12*^*smsk/smsk*^ cells remained unaffected by Ca^++^ or PDMP treatment. Both KLK and protease activity in *smsk* keratinocytes were lower than WT (**, p<0.05). (b) Protease activity was measured in the conditioned media from WT or *Abca12*^*smsk/smsk*^ keratinocytes. Similar to secreted KLK levels, protease activity in WT cells was elevated after Ca^++^ differentiation (p<0.05) and suppressed by PDMP pretreatment. In contrast, protease activity in the cultured media remained unaffected in *Abca12*^*smsk/smsk*^ keratinocytes either after differentiation or PDMP treatment. Sample size n = 3 was used for each condition tested.

The concomitant defects in GlcCer transport and decreased detection of desquamation enzymes in the *smsk* mutant epidermis prompted us to explore whether ABCA12 directly assisted in the transfer of both lipids and KLKs. To test this hypothesis, we followed the fate of fluorescently labelled KLKs and Cer in cultured mouse primary keratinocytes induced to differentiate by elevated Ca^++^ levels. Fluorescent Cer is metabolized into fluorescent sphingomyelin and GlcCer, thus allowing us to study lipid trafficking [39–42]. Under elevated Ca^++^ conditions, extensive colocalization of both KLK5 and -7 with labeled Cer metabolites was observed, indicating that these SC components are cotransported (S3A and S3B Fig). The failure to deliver both GlcCer and the major desquamation enzymes to the SC in our HI mouse model suggested that KLK trafficking could be dependent on GlcCer transport by ABCA12. To test this possibility, we used a highly-specific GlcCer synthase inhibitor, d,l-threo-1-phenyl-2-decanoylamino-3-morpholino-1-propanolhydro- chloride (PDMP), which has been shown to decrease the intracellular levels of GlcCer in both human and mouse keratinocytes in vitro [43, 44]. In WT keratinocytes, the levels and the enzymatic activities of KLK5 and -7 were markedly decreased in the culture media after PDMP treatment (Fig 6), suggesting that de novo glucoceramide production is necessary for proper secretion of KLKs. Intriguingly, the secretion and activity of KLKs from *Abca12*^*smsk/smsk*^ keratinocytes was significantly lower under all culture conditions and was not enhanced by Ca^++^ treatment, presumably due to the inability of the ABCA12 mutant protein to transfer GlcCer (Fig 6A and 6B).

### *Abca12*^*smsk/smsk*^ mouse skin as a model for therapeutic discovery

**Fig 7:**
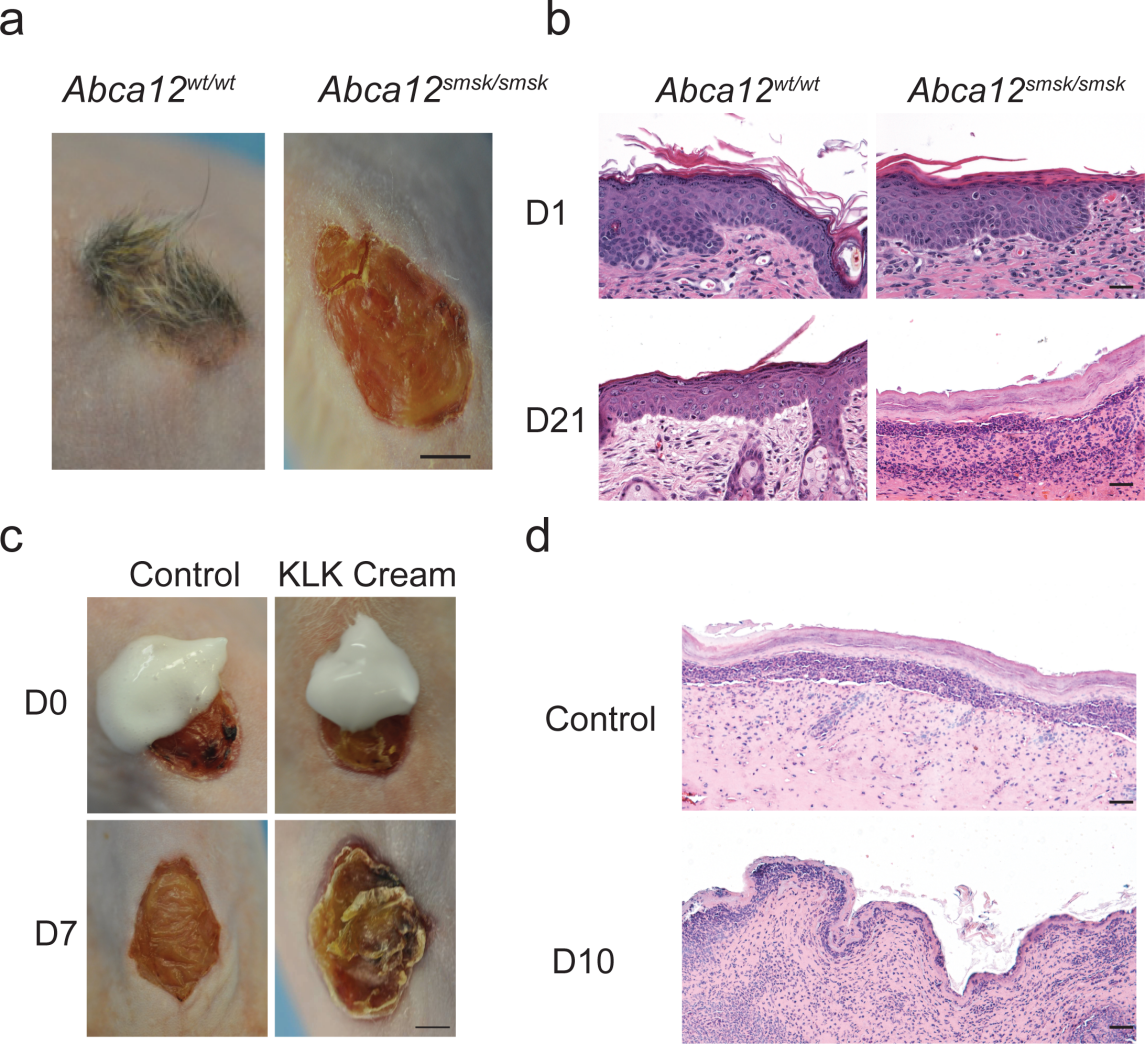
Transplant of *Abca12*^*smsk/smsk*^ skin grafts. (a) Back skins of E18.5 WT and *Abca12*^*smsk/smsk*^ embryos were transplanted onto nude mice. Bar = 2 mm. (b) Histological presentation of H&E stained sections of the grafts from WT and *smsk* transplanted skin. Note the loss of normal epidermal architecture at the end of 3 wks of transplantation in the *smsk* grafts. Bar = 50 μm. (c) *Smsk* E18.5 skin grafts were treated daily with a cream containing either the recombinant KLKs or the digestion buffer (Control). Shedding of the top layers of the hyperkeratotic skin grafts was observed only in grafts treated topically with KLK cream. Images were taken after 7 days of treatment. Bar = 2 mm. (d) Representative H&E images are shown of control and KLK cream treated *smsk* mutant skin grafts. Bar = 10 μm.

The severe desquamatory defects seen in the epidermis of our *smsk* model suggested a possible new treatment strategy to alleviate the hyperkeratosis in HI skin, namely by the topical application of KLK enzymes. To test the feasibility of this strategy we utilized a whole skin graft system as *Abca12*^*smsk/smsk*^ mutant mice die shortly after birth. To accomplish these experiments, we grafted either WT or mutant E18.5 skin onto the back of immunocompromised mice. The grafted *smsk* skin persisted up to one month and maintained the prominent hyperkeratosis and gross architectural characteristic of HI (Fig 7A). However, the mutant skin did not maintain its early post-natal organization (Fig 7B). This was not unexpected as *smsk* skin has a severe barrier dysfunction. Here we focused on whether the application of topical desquamatory enzymes could have an effect on SC thickness and cohesion of HI mutant skin. We applied once daily either a Vaseline-based control cream or an identical cream formulated to contain recombinant KLK 5 and -7 to HI grafts (Fig 7C). In the presence of the KLKs, the top layers of HI grafts started to peel as early as 5 days after treatment, followed by obvious sloughing of the hyperkeratotic SC 7 days after application of the enzymes (Fig 7C and 7D). In contrast, there was no alteration in the appearance of the vehicle treated HI control samples (compare the similar appearance of the *smsk* transplant in Fig 7A with d7 control treated *smsk* sample in Fig 7C). These experiments indicate that the *smsk* model may be used in preclinical studies to pursue novel therapies to ameliorate the severe skin defects of HI newborns.

## Discussion

We have identified a novel mutation in *Abca12* that elicits many of the features of HI, including severe hyperkeratosis, LB defects, absence of intercellular lipid lamellae, aberrant protein processing, defective lipid trafficking, limb contractures and the absence of skin barrier function. Most importantly, this HI mouse model allowed us to uncover the pathophysiological basis for the severe desquamation defects observed in HI and test a novel therapeutic approach for treating HI and potentially other hyperkeratotic disorders.

One of the most pronounced features of HI is the striking hyperkeratosis of the epidermis. A mechanistic understanding of this pathological feature of HI has remained elusive [30, 45]. In our studies, CDSN, whose deficiency has been implicated with flaky skin in humans [46] and mice [47], was markedly reduced in the *Abca12*^*smsk/smsk*^ SC. However, it is clear that the reduced levels of CDSN is secondary to the pronounced cohesion between corneocytes in mutant skin and indicates that the maturation of desmosomes into CDs may also be defective. The reduced lipid lamellar structures observed in the SC interstices of the mutant epidermis contribute to the tight packing of corneocytes. Nevertheless, we present several lines of evidence indicating that the hyperkeratosis observed in HI is likely caused by a severe defect in desquamation. First, proliferative rates in basal layer keratinocytes were unaltered in *Abca12*^*smsk/smsk*^ skin, discounting enhanced proliferation as a cause for hyperkeratosis. Second, corneocytes in *Abca12*^*smsk/smsk*^ SC were highly resistant to chemical dissociation. Third, CDs were retained in the SC of *Abca12*^*smsk/smsk*^ epidermis, while desquamation enzymes KLK5 and -7 were absent or reduced. Lastly, exogenous application of KLKs induced flaking in grafts from *Abca12*^*smsk/smsk*^ skin, which mimicks the normal process of desquamation. In support of our findings, previous studies have shown defects in the delivery of proteolytic enzymes (e.g., KLK7) and other LB cargo proteins to the SC in human HI tissue [48, 49], and the secretion of KLK5 is diminished in *Abca12*-null mouse keratinocytes [50]. Other groups have reported the persistence of CDs in the distal layers of the epidermis in ABCA12 mutant mice [23, 25]. Moreover, mice deficient in INVOLUCRIN, ENVOPLAKIN, and PERIPLAKIN show barrier defects, decreased desquamation, and hyperkeratosis that is concomitant with decreased KLK activity [51]. These observations and our current findings strongly argue that HI is not simply a defect caused by aberrant lipid trafficking, but also a disease of defective desquamation.

To address how defects in lipid trafficking may alter desquamation, we performed a series of experiments to determine whether ABCA12 may be required for the transport of lipids and KLKs. We showed that Cer and its metabolites (e.g., GlcCer) colocalized with KLKs in differentiating keratinocytes and that inhibition of intracellular GlcCer production led to a reduction in KLK accumulation in the media of cultured keratinocytes. These findings agree with previous studies that showed a strong link between lipid and enzyme accumulation in LBs [45]. Our data indicate that ABCA12 may function to directly transport lipids, thereby providing LBs with structural properties that enable the uptake and transport of KLKs. Alternatively, ABCA12 might be independently required for the transport of both lipids and enzymes. Further analysis will be required to distinguish between these possibilities. The *Abca12*^*smsk/smsk*^ mouse model results from a splice site mutation that causes exon skipping and loss of coding sequences from the first ATP binding cassette. The *smsk* mutant model, as is the case for previously described ABCA12 mutant mice [23–25] would be expected to act as a null mutation and cause the severe skin phenotype seen in HI patients. Other mutations in *ABCA12* result in the less severe skin diseases, LI 2 and NBCIE [19, 20]. It will be important to determine whether lipid and/or protein transport through LBs is also defective in these patients.

In HI, it is now apparent that loss of ABCA12 function prevents the transfer of lipids and KLKs through the LBs, thus impairing both lipid barrier formation and desquamation. Therefore, HI is not simply a disease of deregulated lipid metabolism in the epidermis, but also a disease of profound desquamation defects. Current treatments for HI and other diseases of the ARCI spectrum include treatment with retinoids and skin softeners, but these are limited in preventing disease progression. Therefore, we utilized the new pathogenic insights gained from our *Abca12*^*smsk/smsk*^ HI studies to test an alternative treatment strategy by the topical application of enzymes involved in desquamation. We demonstrated that topical application of recombinant KLKs can efficiently alleviate the severe hyperkeratosis that develops in transplanted HI mouse skin grafts. These results further strengthen our conclusion that the absence of proteolytic enzymes is a major factor contributing to the pathogenesis of HI, and also suggest that the topical application of desquamation enzymes may represent a novel therapeutic strategy for the treatment of HI and potentially other hyperkeratotic disorders.

## Materials and Methods

### Animal, DNA and RNA experiments

Animal experiments were approved by The University of Colorado Institutional Animal Care and Use Committee (IACUC). The ENU screen was performed as previously described [52]. Heterozygous carriers were mated and the embryonic stages were determined by considering noon of the day of a vaginal plug as E0.5. Embryonic skins were either directly embedded in OCT followed by freezing on dry ice or fixation in 4% paraformaldehyde for paraffin processing. RNA isolation and cDNA production were performed using standard techniques and as previously described [53]. *Abca12* cDNA fragments encompassing exons 28 to 31 (764bp) were amplified by RT-PCR using the following oligos: forward– 5’- gtc ctg act gtg cat ttc cct cca aca-3’, and reverse– 5’-ttc ttc ttg gtg agt gtg agg tgg ta-3’. PCR products were then resolved and visualized on 1.2% agarose gel containing ethidium bromide. The cDNA fragments were isolated and analyzed by dideoxy-sequencing. *Abca12* expression was evaluated using Taqman probe sets for *Abca12* and *Gapdh* (Abca12:Mm00613683_m1; Gapdh:4351309). *Klk7* expression was evaluated using Sigma Aldrich KiCqstart predesigned primers: *Klk7*-Fw: 5’-agg aga aag gat tat aga tgg c -3’ & *Klk7*-Rv: 5’-ctt gct act gac cca ttt tg -3’). *Cdsn* expression was evaluated using a predesigned Primetime probe through IDT (Mm.PT.58.6164239) and normalized to the Taqman probe for *Gapdh* mentioned above. All assays were run on a Roche LightCycler480 and fold change determined by the ΔΔCt method.

### Immunofluorescence staining

Immunofluorescence was performed as previously described [53]. Sections were incubated with antibodies against KRT -1, -8, -6, -14, and -16, FILAGGRIN, and LORICRIN [54], KLK5 (H-55; Santa Cruz Biotechnology, Dallas, Texas), KLK7 (H-50; Santa Cruz Biotechnology) anti-GlcCer/Cer (RAS_0011; Glycobiotech, Bostel, Germany), anti-Desmoplakin (Fitzgerald; Acton, MA), anti-DSC3 (U114; Progen; Heidelberg, Germany), anti-DSG1/2 (DG3.10; Progen), anti-Involucrin (M-116; Santa Cruz Biotechnology) and anti-CDSN (P-20; Santa Cruz Biotechnology). The anti-GlcCer/Cer antisera shows cross-reactivity in both the SC and SG [50, 55, 56]. Alexa-fluor conjugated secondary antibodies were purchased from Invitrogen. For determination of proliferation index, pregnant mice were injected i.p. with BrdU (Invitrogen -Thermo Fisher Scientific, Waltham, MA) and sacrificed 2h after injection. Embryos were harvested and processed for histology. Slides were stained with an anti-BrdU antibody (Invitrogen). Nuclei were counterstained with DAPI (Vector Laboratories, Burlingame, CA). Image acquisition for all staining experiments was performed on a Nikon 90i microscope system (Nikon Corporation, Tokyo, Japan)

### Skin permeability assay and cornified envelope preparations

Toluidine blue dye penetration assays were performed as previously described [57]. CEs and epidermal protein samples were prepared as previously reported without sonication steps [58]. Embryo skin pieces (0.25 cm^2^) were boiled at 95°C for 10 min in extraction buffer (0.01 M Tris pH 7.4, 2% SDS, 100 mM NaCl, 5 mM EDTA pH 8.0).

### Transmission Electron Microscopy

Mid-dorsum skins were processed for TEM as described with minor modifications [45, 59]. Briefly, all samples were postfixed in reduced 1% osmium tetroxide containing 1.5% ferrocyanide in 0.1 M cacodylate buffer followed by rinsing and en bloc staining in 2% aqueous uranyl acetate. The samples then were dehydrated in a graded ethanol series and embedded in Epon. Sections were cut on a Leica Ultracut E and observed on a Zeiss 10A electron microscope (Carl Zeiss Inc, Thornwood, NY) at 60 kV without further counterstaining.

### Localization of labeled KALLIKREIN proteins and C5 ceramides in primary cultures of mouse keratinocytes

Recombinant human KLK5 (R&D Systems, Minneapolis, MN) and KLK7 (Invitrogen) were labeled with Alexa Fluor 488 Protein Labeling Kit per manufacturer’s instructions (Invitrogen). Labeled KLKs were transfected into cultured WT keratinocytes using X-fect Protein Transfection Reagent (Clontech, Mountain View, CA) and then incubated with BODIPY TR- labeled ceramides (Invitrogen). Cells were then cultured for 12h in 0.05mM Ca^++^, and then induced to differentiate by increasing Ca^++^ levels in the media to 0.6mM.

### Determination of secreted KALLIKREIN proteins and protease activity from cultured keratinocytes

For these experiments, we used primary keratinocytes cultures grown in low and high calcium conditions (see above). In KLK Elisa assays, 96-well plates (Corning, Corning, NY) were coated with rabbit anti-KLK5 or KLK7 antibody (Santa Cruz Biotechnology) at 1 μg/ml in phosphate-buffered saline (PBS). The wells were washed, blocked with PBS containing 1% BSA, and then incubated for 3 h with media isolated from keratinocytes grown for 12 hours in fresh media. Biotinylated goat anti-KLK5 or anti-KLK7 antibodies (R&D Systems) were used as detection antibodies. Colorimetric quantification at 450 nm was carried out by incubating plates with streptavidin-conjugated horseradish peroxidase (R&D Systems, Minneapolis, MN) and 3,3′5,5′-tetramethylbenzidine substrate (BD Biosciences, San Jose, CA). The reactions were stopped by 0.2 M sulfuric acid (Sigma-Aldrich, St. Louis, MO). The enzymatic activity of KLK proteins was measured by an EnzChek Protease Assay Kit (green fluorescence, Invitrogen) following the manufacturer’s recommendations. Absorbance or fluorescence was measured on a Glomax plate reader (Promega, Madison, WI)

### Skin transplantation and KALLIKREIN treatment

Skin transplantation procedures were performed as described with modifications [60]. Briefly, E18.5 back skin biopsies (>2.0 cm^2^) of *Abca12*^*smsk/smsk*^ or WT embryos were transplanted onto the back of nude mice under anesthesia (Isoflurane). Mice were treated with Carprofen [5 mg/kg] prior to surgery and 24 hours post-surgery, totaling 48 hours of analgesia. The whole transplanted area was covered with sterile dressing for 24h and then left uncovered for the duration of the experiment. Mice were sacrificed using a high dose of Isoflurane in accordance with The University of Colorado IACUC. Recombinant human KLK5 (R&D Systems) and KLK7 (Invitrogen) proteins were diluted in the digestion buffer (100mM sodium phosphate, 0.1mM EDTA, pH 7.4.0) at working concentrations. KLKs were then formulated into a Vaseline-based cream by diluting KLKs into 1x digestion buffer at a final concentration of KLK5 [5ng/uL] and KLK7 [10ng/uL]. 1 mL of the enzyme solution was combined with 1mL unscented Vaseline and vortexed at maximum speed for 1hr or until homogeneous. Starting from day 3 post-transplantation, the *Abca12*^*smsk/smsk*^ skin grafts were topically treated with cream containing KLK5 and -7, or the digestion buffer only as a control. HI grafts were harvested after 7-21d of daily applications.

### Statistics

Experiments were repeated independently at least twice with n = 3 replicates for each condition. A student’s t-test was used to determine significance using Graph pad Prism v.5 software.

## Supporting Information

S1 Fig Retention of corneodesmosomes in *Abca12*^*smsk/smsk*^ skin. (a) TEM of the SC was performed on E18.5 WT and *smsk* mutant skin samples. The image in WT shows the lower SC and underlying granular layer (SG). The image of the mutant is at the same magnification as the WT, showing the lower layers of the SC. Arrows denote CDs. In WT skin, CDs are evident in the lower SC, the first two layers of SC closest to the SG. Bar = 1 μm. Note the persistence of CDs throughout the *smsk* mutant SC. Moreover, *smsk* mutant skin exhibited incomplete (delayed) cornification with corneocytes appearing transitional. Bar = 1 μm. (b) CD density was quantified by counting the number of CDs per length of corneocyte membranes in the field. *Smsk* skin had significantly more CDs than WT skin both in the lower and upper SC, which verified the retention of CDs in mutant SC. The values shown represent the average ± SD of 10 images from n = 3 embryos analyzed per genotype.
(TIF)

S2 Fig Gene expression analysis of *Kallikreins* and *Corneodesmosin* in *Abca12*^*smsk/smsk*^ skin. qPCR analysis was performed on RNA isolated from E18.5 WT and *smsk* skin. Expression was normalized to *Gapdh*. *Abca12*, *Klk7* and *Cdsn* transcripts were significantly upregulated in mutant samples.
(TIF)

S3 Fig KALLIKREIN proteins colocalize with ceramide metabolites in differentiating keratinocytes. **(a)** Recombinant KLK5 was labeled with Alexa 488 (green), and C5-ceramides with Bodipy-TR (red), and were introduced into cultured WT mouse keratinocytes. Ceramides are metabolized into GlcCer in keratinocytes. After inducing differentiation, KLK5 was extensively co-detected with labeled lipids. Similar results were observed with KLK7 (not shown). Bar = 10 μm. (b) Colocalization of labeled lipids and KLKs was determined by confocal microscopy. Images shown represent 1 micron steps covering the depth of a single stained keratinocyte. Note the merging of the green and red staining (yellow granules) in the images. Bar = 5 μm.
(TIF)
