# Supplementary figures and images for: Defects in Stratum Corneum Desquamation Are the Predominant Effect of Impaired ABCA12 Function in a Novel Mouse Model of Harlequin Ichthyosis
Source: PLoS One. 2016 Aug 23;11(8):e0161465. doi: 10.1371/journal.pone.0161465 (PMC4994956; doi:10.1371/journal.pone.0161465)

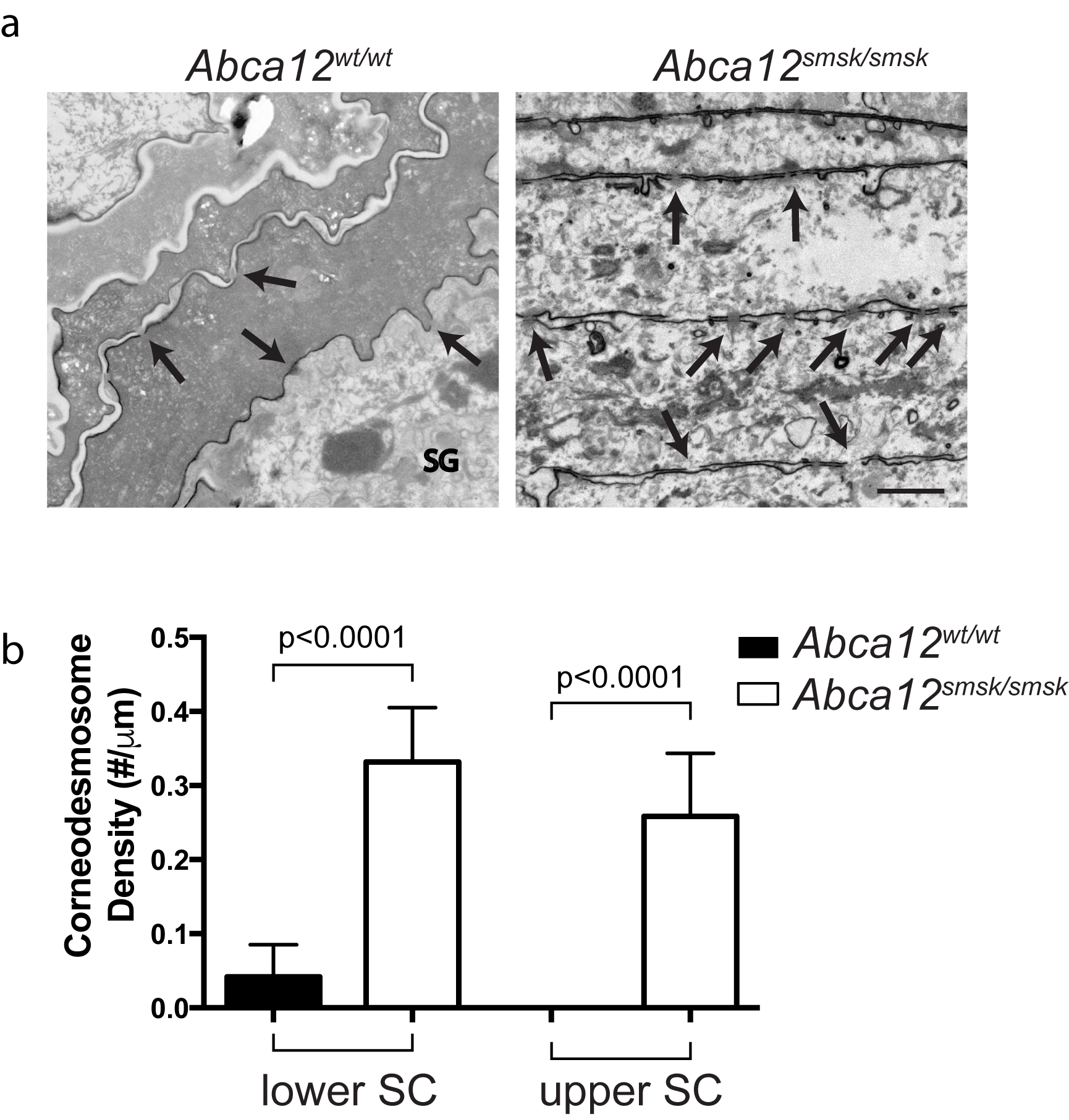

Supplement: S1 Fig — (a) TEM of the SC was performed on E18.5 WT and smsk mutant skin samples. The image in WT shows the lower SC and underlying granular layer (SG). The image of the mutant is at the same magnification as the WT, showing the lower layers of the SC. Arrows denote CDs. In WT skin, CDs are evident in the lower SC, the first two layers of SC closest to the SG. Bar = 1 μm. Note the persistence of CDs throughout the smsk mutant SC. Moreover, smsk mutant skin exhibited incomplete (delayed) cornification with corneocytes appearing transitional. Bar = 1 μm. (b) CD density was quantified by counting the number of CDs per length of corneocyte membranes in the field. Smsk skin had significantly more CDs than WT skin both in the lower and upper SC, which verified the retention of CDs in mutant SC. The values shown represent the average ± SD of 10 images from n = 3 embryos analyzed per genotype. (TIF) [file pone.0161465.s001.tif]

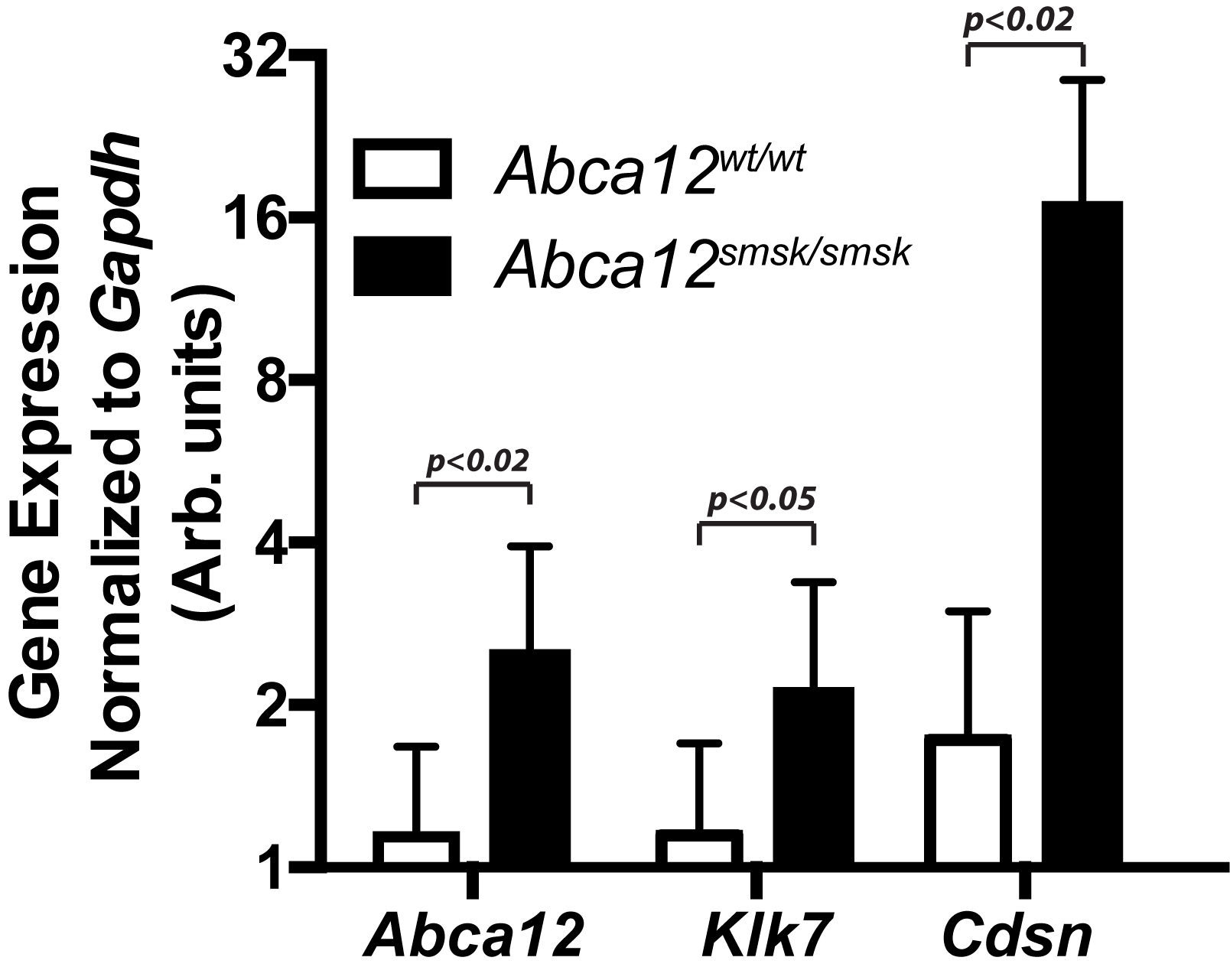

Supplement: S2 Fig — qPCR analysis was performed on RNA isolated from E18.5 WT and smsk skin. Expression was normalized to Gapdh. Abca12, Klk7 and Cdsn transcripts were significantly upregulated in mutant samples. (TIF) [file pone.0161465.s002.tif]

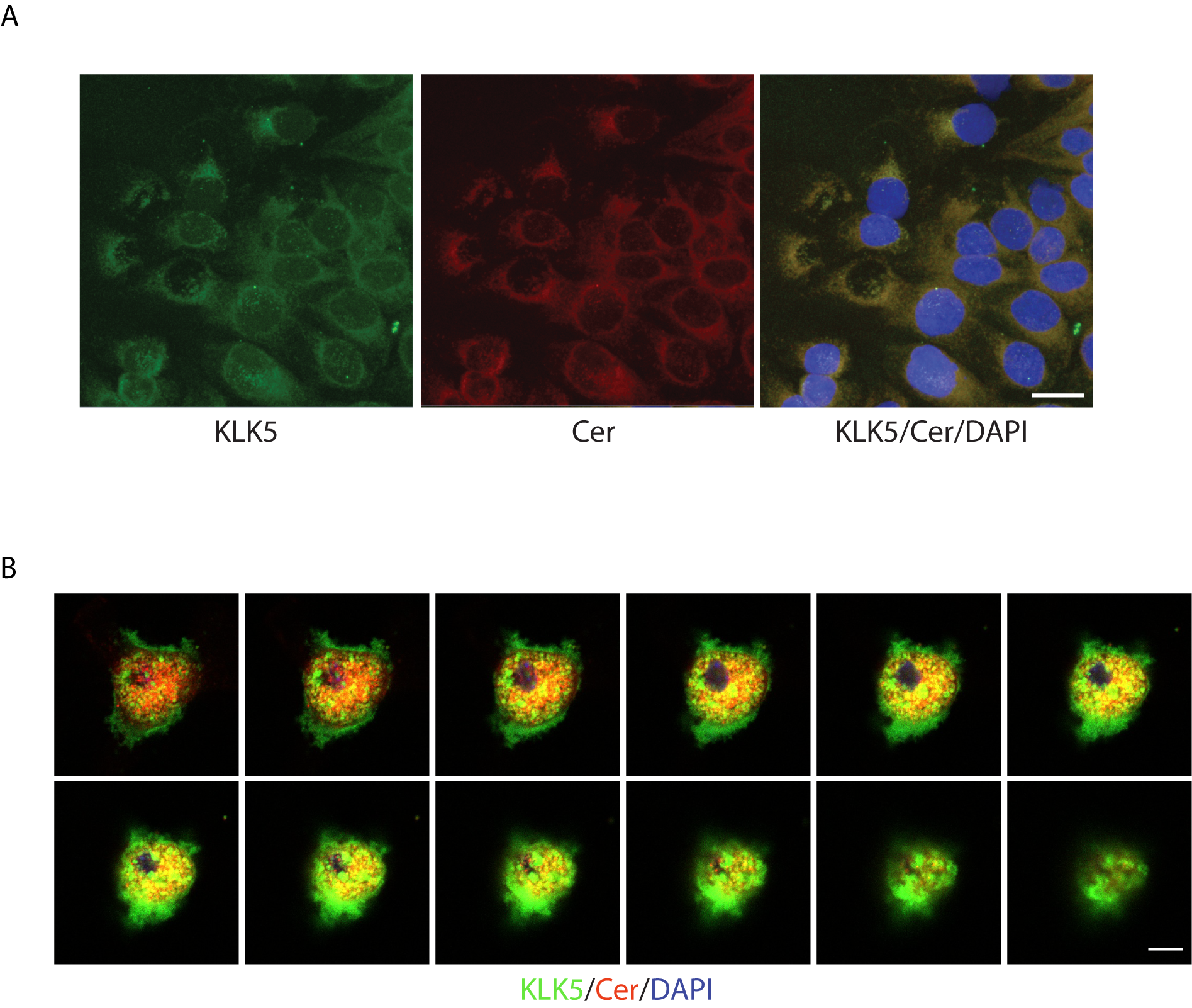

Supplement: S3 Fig — (a) Recombinant KLK5 was labeled with Alexa 488 (green), and C5-ceramides with Bodipy-TR (red), and were introduced into cultured WT mouse keratinocytes. Ceramides are metabolized into GlcCer in keratinocytes. After inducing differentiation, KLK5 was extensively co-detected with labeled lipids. Similar results were observed with KLK7 (not shown). Bar = 10 μm. (b) Colocalization of labeled lipids and KLKs was determined by confocal microscopy. Images shown represent 1 micron steps covering the depth of a single stained keratinocyte. Note the merging of the green and red staining (yellow granules) in the images. Bar = 5 μm. (TIF) [file pone.0161465.s003.tif]
